# GRADE guidance 24 optimizing the integration of randomized and non-randomized studies of interventions in evidence syntheses and health guidelines

**DOI:** 10.1016/j.jclinepi.2021.11.026

**Published:** 2022-02

**Authors:** Carlos A. Cuello-Garcia, Nancy Santesso, Rebecca L. Morgan, Jos Verbeek, Kris Thayer, Mohammed T. Ansari, Joerg Meerpohl, Lukas Schwingshackl, Srinivasa Vittal Katikireddi, Jan L. Brozek, Barnaby Reeves, Mohammad H. Murad, Maicon Falavigna, Reem Mustafa, Deborah L. Regidor, Paul Elias Alexander, Paul Garner, Elie A. Akl, Gordon Guyatt, Holger J. Schünemann

**Affiliations:** a Department of Health Research Methods, Evidence, and Impact, McMaster University, Hamilton, Ontario, Canada; b Department of Health Quality Improvement. Tecnologico de Monterrey School of Medicine, Monterrey, Nuevo Leon, Mexico; c Cochrane Work Review Group. Occupational Medicine. Finnish Institute of Occupational Health, Helsinki, Uusimaa, Finland; d Integrated Risk Information System (IRIS) Division, National Center for Environmental Assessment, Environmental Protection Agency, Washington, D.C., USA; e School of Epidemiology and Public Health, Faculty of Medicine, University of Ottawa, Ottawa, QC, Canada; f Institute for Evidence in Medicine, Medical Center & Faculty of Medicine, University of Freiburg, Freiburg, Baden-Wurttemberg, Germany; g Cochrane Germany, Cochrane Germany Foundation, Freiburg, Baden-Wurttemberg, Germany; h MRC/CSO Social & Public Health Sciences Unit, University of Glasgow, Greater Glasgow, Scotland; i Department of Medicine, McMaster University. Hamilton, Ontario, Canada; j School of Clinical Sciences, University of Bristol, Bristol, South West, England; k Evidence-Based Practice Center, Mayo Clinic, Rochester, Minnesota, USA; l Federal University of Rio Grande do Sul, Institute for Health Technology Assessment, Porto Alegre, Rio Grande do Sul, Brazil; m Division of Nephrology and Hypertension, Department of Medicine, University of Kansas Medical Center, Kansas City, Missouri USA; n Evidence Services, Kaiser Permanente, Care Management Institute, Oakland, California, USA; o Department of Clinical Sciences, Liverpool School of Tropical Medicine, Liverpool, Merseyside, England; p Department of Internal Medicine, American University of Beirut, Beirut Governorate, Lebanon

**Keywords:** GRADE, Quality of evidence, Certainty of evidence, Risk of bias, Non-randomized studies, ROBINS

## Abstract

**Background and Objective:**

This is the 24th in the ongoing series of articles describing the GRADE approach for assessing the certainty of a body of evidence in systematic reviews and health technology assessments and how to move from evidence to recommendations in guidelines.

**Methods:**

Guideline developers and authors of systematic reviews and other evidence syntheses use randomized controlled studies (RCTs) and non-randomized studies of interventions (NRSI) as sources of evidence for questions about health interventions. RCTs with low risk of bias are the most trustworthy source of evidence for estimating relative effects of interventions because of protection against confounding and other biases. However, in several instances, NRSI can still provide valuable information as complementary, sequential, or replacement evidence for RCTs.

**Results:**

In this article we offer guidance on the decision regarding when to search for and include either or both types of studies in systematic reviews to inform health recommendations.

**Conclusion:**

This work aims to help methodologists in review teams, technology assessors, guideline panelists, and anyone conducting evidence syntheses using GRADE.

## Highlights

• Randomized controlled trials (RCTs) provide the best source of evidence for research syntheses estimating relative effects of an intervention.
• Non-randomized studies of representative populations can provide the best evidence with respect to prognosis, baseline risk, test accuracy, and estimates of utility and values and preferences of different outcomes.
• For many research questions randomized trials will be scarce or unavailable, and decision-makers might need to consider using non-randomized (observational) studies that can provide evidence about the effectiveness of interventions as replacement (in the absence of appropriate RCT evidence), sequential, or complementary to RCT evidence
• GRADE guidance can help authors that are considering the inclusion of non-randomized studies in addition to RCTs during the evidence synthesis process.

**What is new?**

**Key findings**
• GRADE provides guidance for deciding when to use different types of individual studies to be included in evidence synthesis of health interventions, whether authors consider RCTs or NRSI.

**What this adds to what is known**
• Using a framework that considers the certainty of evidence of randomized and non-randomized studies, first separately and then in an integrative fashion, can help with the decision to include one or both types of studies in evidence syntheses.

**What is the implication, what should change now**
• This GRADE guidance will help increase the certainty and comprehensiveness of a body of evidence to answer a question about a health intervention and improve recommendations by considering different types of study designs.

Alt-text: Unlabelled box

## 1 Background

Randomized controlled trials (RCTs) provide the best source of evidence for research syntheses estimating relative effects^1^ of an intervention that might inform health guidelines. Non-randomized studies of representative populations can provide the best evidence with respect to prognosis, baseline risk, test accuracy, and estimates of utility and values and preferences of different outcomes [1], [2], [3]. Non-randomized studies may also provide evidence about the effectiveness of interventions as replacement (in the absence of appropriate RCT evidence), sequential, or complementary to RCT evidence (see box 1) [4]. While non-randomized studies of interventions (NRSI) may potentially provide more generalizable or precise evidence compared to RCTs, confounding and other biases restrict their use [5].

**Table:** 

**Box 1. Potential role of NRSI in evidence syntheses**

**Complementary** — NRSI provide additional information on: • whether or not an intervention works in different populations that one wants to extrapolate to (e.g., NRSI studies provide evidence for populations not included in RCTs). • possible effect modification (e.g., NRSI provide complementary evidence that lend support to evidence from RCTs that suggests or refutes effect modification) • estimates of baseline risk in different non-trial settings **Sequential** — NRSI provide information that is not (yet) obtained or available from RCTs on: • long-term or rare (beneficial and/or harm) outcomes • correlation between surrogate outcomes and patient important outcomes **Replacement** — NRSI are used instead of RCT evidence for decision making because: • NRSI provide higher certainty evidence than RCTs (this applies when NRSI provide more direct and/or precise evidence that leaves us with greater overall confidence in estimates of effect or certainty of evidence). • RCTs are absent and NRSI provide the best available evidence

Authors of evidence syntheses of health interventions aim for the highest certainty evidence, and guideline developers need these syntheses to generate trustworthy recommendations. This explains the advisability of incorporating evidence from NRSI in systematic reviews when they provide complementary, sequential, or replacement evidence to RCTs [6,7].

If RCTs alone may be unable to answer a PICO question (population, intervention, comparison, and outcome) an evidence review team faces the following issues: (a) When to search and include both types of study designs? (b) What are the optimal methods to synthesize information from both type of studies, including the decisions about pooling data from different study designs? (c) How should authors present results in evidence profiles and summary of findings tables? and (d) When both RCTs and NRSI contribute to the evidence synthesis, what is the possible influence on certainty of the evidence? Interpretation issues may be particularly challenging when RCTs and NRSI show differences in the direction or magnitude of effects, as well as in other individual GRADE domains. This 24th article in the ongoing GRADE series in the Journal of Clinical Epidemiology represents the GRADE Working Group guidance assessing the first question: that is, when is it appropriate to search for and include NRSI in addition to RCTs during the evidence synthesis process.

## 2 Methods and outline

This guidance is based on previous published work [4,6,8], scoping reviews, and surveys of experts and members of Cochrane and the Guidelines International Network (GIN). We used an iterative approach to develop and refine the concepts addressed in this guidance during face-to-face and online meetings with members of the GRADE Risk of Bias in Non-randomized Studies Risk of Bias Project Group specifically, and the GRADE Working group more broadly. In the first section, we will consider reasons for integrating NRSI at the early stages of formulating a research question for an evidence synthesis. The second section presents possible scenarios encountered when evaluating a body of evidence (for a given outcome) that includes RCTs and NRSI. Finally, we discuss future areas of research.

For this guidance, we define **evidence synthesis** as any systematic review, rapid review, health technology assessment or any other method aiming to summarize the evidence with the highest certainty available for a specific question about the effects of an intervention [9]. We address the perspectives of both evidence synthesis authors and guideline developers — who aim to produce recommendations. Although we will at times mention that NRSI are ideal for assessing baseline risk, our focus is primarily on the use of NRSI to estimate relative effects of health interventions and technologies (e.g., medications, behavioral interventions, devices). Lastly, when we use the term “integration” it will broadly refer to any form of using RCT and NRSI together, either in the same synthesis, or in the same summary of findings (same table but separated in rows).

## 3 When to include and search for non-randomized studies in evidence syntheses of interventions?

### 3.1 The role of a protocol and search strategy

**Fig. 1:**
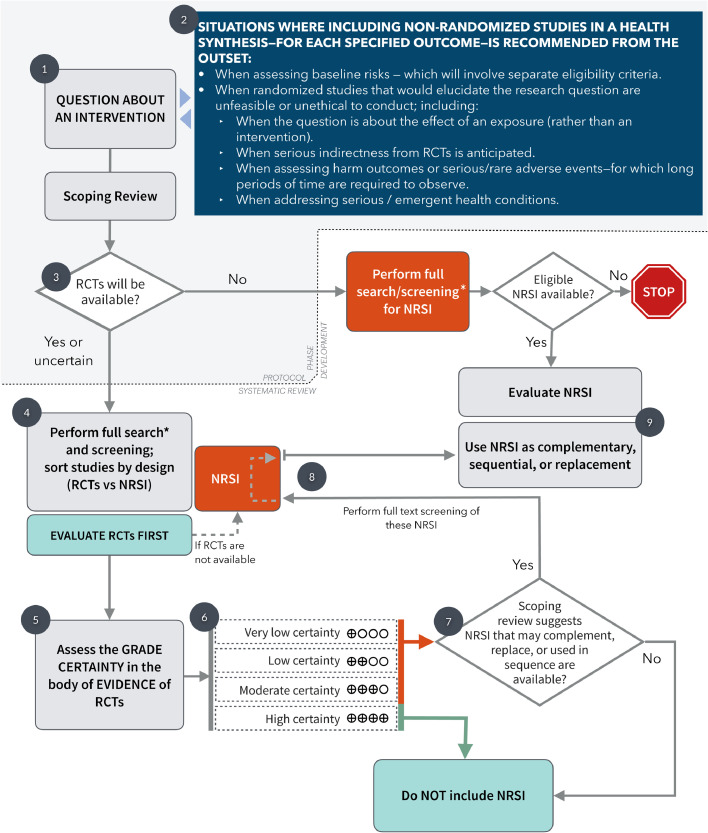
Flowchart depicting the process of conducting a systematic review about a health intervention considering the role of randomized and non-randomized studies. The explanations are portrayed as dark grey circles. *See text* for detailed description of each step. The grey area represents the steps that might be taken during the protocol development. Of note, as stated in the GRADE guidance, the assessment of the certainty of evidence should be made for each outcome evaluated in the review process. *In most situations, the search strategy performed for the scoping review will be comprehensive enough to be used at this step; however, authors may prefer to run another search or include changes from the one in the scoping review.

Fig. 1 depicts a flowchart of the proposed steps to incorporate RCTs and NRSI in an evidence synthesis. It is important for authors to detail from the outset (i.e., at the protocol stage [at Point #1 in Fig. 1]) any pre-specified criteria about the design of the studies they will search for (RCT, NRSI, or both) and the circumstances in which they will include these studies.

Guideline developers and authors of systematic reviews may have reasons to search for and include NRSI from the outset — this is, other than for assessing relative effects of interventions — irrespective of the availability of RCTs (Point #2 in Fig. 1). For instance, a common reason would be to address baseline risks, or to assess interventions and outcomes for which RCTs are unfeasible, unethical to conduct, sparse or unavailable (e.g., rare adverse outcomes or in emergent conditions), or when authors anticipate very serious indirectness in the evidence from RCT. This guidance will focus on the common situation when review authors have reasons to believe that NRSI will provide complementary, sequential, or replacement information that will make important contributions to the overall certainty of evidence.

The review team, before finalising the protocol (depicted in Fig. 1 as the shaded area), must scope the available evidence and specify the criteria for the best study designs for different research (PICO) questions [10]. The scoping review, as precursor of the systematic review during the protocol stage, allows authors to detect and describe appropriate synthesis methods, analyze the gaps in the knowledge base, and facilitate estimating the amount of work and resources needed to complete the review [11,12]. The specific type of NRSI to be included should not rely on classic 'evidence hierarchies' for studies of effectiveness, but rather on an assessment of the individual PICO question and the eligibility criteria; this is, the best NRSI design that is likely to be available and provide the highest certainty of evidence [13].

The scoping review will inform the reviewers if RCTs are likely to be available, or if there is some uncertainty around the issue (Point #3 in Fig.1); this decision, once is resolved, should be established in the protocol, as well as any other inclusion criteria. Once the protocol is accepted, the following step is a full, sensitive literature search with screening of titles and abstracts that will include both types of studies (RCTs and NRSI) for the research question (point #4, Fig. 1). In most situations, the search strategy from the scoping review will be comprehensive enough to be used for the full systematic review, and a single search strategy will be sufficient.

The references obtained from the literature search can be sorted by study design (first RCTs, then NRSI). Current reference managers and filters can make this sorting process feasible[14,15]. At this stage (point #4 in Fig. 1), authors can, after screening titles and abstracts, separate RCTs from NRSI; if RCTs are found, reviewers may proceed to extract data and assess the risk of bias and GRADE the certainty of the evidence (points #4 and #5 from Fig. 1). However, if RCTs are not available, reviewers will proceed to evaluate the NRSI found from the search strategy.

This process requires a review team with the appropriate content and methodological expertise, with a protocol describing precise methods for the optimal use of RCTs and NRSI. Although screening and reviewing NRSI are more time consuming than for RCTs [11], a strategy that includes and sorts all likely study designs is efficient to attain the most comprehensive and appropriate body of evidence. We stress the requirement to have an information specialist in the team with expertise in systematic reviews and scoping reviews[16,17]. At this point, experts and the review team may be certain that there is either sufficient RCT evidence to address relative effects for all important outcomes or, alternatively, that there is no RCT evidence for one or more patient-important outcomes and should move to only use NRSI.

### 3.2 When to include non-randomized studies

After completing the sorting process and data extraction and risk of bias evaluation of available RCTs, authors should use the GRADE methodology to assess each RCT. Importantly, this assessment should always be made considering each outcome to rate the certainty of the body of evidence (points #5 and #6 of Fig. 1). If authors conclude there is high certainty of evidence from RCTs, further evaluation of NRSI to complement estimates of relative effect for that outcome will not be necessary and authors can use only the evidence from RCTs. We emphasise that high certainty evidence for some important outcomes (typically benefit) provides no guarantee of high certainty evidence for other important outcomes (typically rare harms) [10,11] and for this reason this process should always be considered for each outcome separately.

We have identified 2 general scenarios when there is no high certainty evidence from RCTs (within Point #6, Fig. 1): First, in situations where RCT evidence is deemed low, or very low certainty, NRSI may help increase the overall level of certainty. Reviewers should search for and evaluate NRSI, if they consider it plausible that NRSI will yield evidence equal or superior to that from the RCTs. In (Table 1) we present an example to visualize NRSI evidence of equal certainty than RCT evidence for an outcome. In this case, similar evidence classified as the same certainty could be useful for decision-making. Second, when certainty of evidence from RCTs is rated moderate, authors should consider integrating NRSI evidence if it could mitigate concerns about indirectness in the RCT evidence. This situation will be more likely to occur when indirectness is present and NRSI evidence serves as complementary or sequential evidence. In this scenario, it will be unlikely to find NRSI categorized as high or moderate certainty that trumps the RCTs, because NRSI can only be correctly classified as such when authors find reasons for rating up (typically–very–large effects or dose response relationships), or (on rare occasions) when assessed as low risk of bias — using an appropriate risk of bias tool, such as ROBINS-I (see below). Large NRSI with precise effect estimates that narrow confidence intervals may be tempting to use; however, caution should be used as they can be misleading if their estimates are biased.

**Table 1:** Evidence profile using randomized and non-randomized studies of interventions for the same outcome and similar certainty of evidence.

Certainty assessment							№ of patients		Effect		Certainty	Importance
№ of studies	Study design	Risk of bias	Inconsistency	Indirectness	Imprecision	Other considerations	Vitamin D	No vitamin D	Relative(95% CI)	Absolute(95% CI)		
Asthma / wheezing—Randomized studies												
1	randomised trials [22]	not serious a	not serious	not serious	very serious b	none	17/108 (15.7%)	7/50 (14.0%)	RR 1.12 (0.50 to 2.54)	17 more per 1,000 (from 70 fewer to 216 more)	⊕⊕◯◯ LOW	CRITICAL
Ashtma / recurrent wheezing—Non-randomized studies												
6	non-randomized studies [22], [23], [24], [25], [26], [27], [28]	very serious c	not serious	not serious	not serious	none d	8,831 e	26,553	OR 0.76 (0.69 to 0.84)	30 fewer per 1,000 (from 39 fewer to 20 fewer)	⊕⊕◯◯ LOW	CRITICAL
								risk: 14.0%				

Question: Vitamin D compared to no vitamin D in pregnant women for preventing asthma or wheezing in their offspring

Setting: ambulatory

CI, Confidence interval; RR, Risk ratio; OR, Odds ratio

Explanations

a There were 22/180 participants who were not analyzed (lost to follow-up), 16% in the intervention group and 10% in the control group. Also, the outcome was a subjective measure and participants were not blinded to treatment allocation (reporter bias).

b Wide confidence interval with a small number of participants for the optimal information size; also, crossing the null and the appreciable thresholds for benefit and harm.

c All studies have bias due to possible residual confounding and bias due to selection of participants. The non-randomized studies thus are downgraded two levels. The ROBINS-I tool was used. No further downgrading was considered necessary.

d All individual studies report a significant dose-response association at various levels of vitamin D supplementation on the risk of asthma or wheezing. This, however, can be due to a spurious effect if residual confounding remains within each study. By visually inspecting the forest plot based on different vitamin D dosages, the effect looks minimal. We decided not to upgrade but if such case were considered, the overall certainty will end as MODERATE.

e All studies provide the adjusted odds ratios on the risk of asthma and its association with vitamin D intake. Baseline risk in the control group was assigned from the rest of the studies, including the randomized controlled trial.

Once it has been decided to use NRSI based on any of the above situations, authors should go back to screen and evaluate the group of references of NRSI that were available from the scoping of the literature (Points #7 and #8, Fig.1) as these may complement, replace, or used in sequence (as explained in Box 1). This would be based on the initial criteria for NRSI established in the protocol (Points #8 and #9, Fig. 1).

## 4 Integrating randomized and non-randomized studies in evidence syntheses

### 4.1 Possible scenarios when dealing with two bodies of evidence

**Fig. 2:**
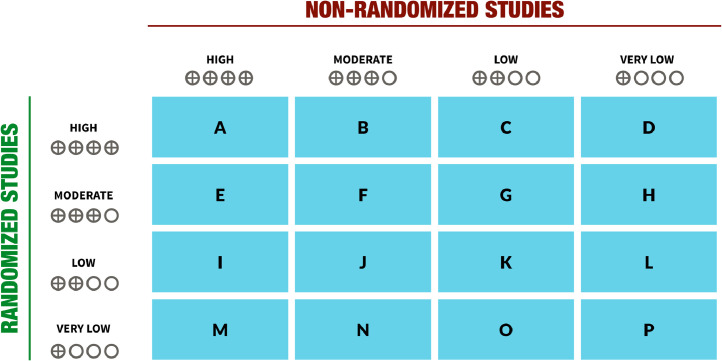
Situations after assessment of the GRADE certainty of evidence when NRSI and RCTs are included in an evidence synthesis. See also Point 9 from Figure 1 and full description within the text.

In Fig 2 we present the possible scenarios that can arise when bodies of evidence of RCTs and NRSI may answer the same health question for a specific outcome. Although 16 combinations are theoretically possible, looking for NRSI is not necessary in some situations; for instance, for cells A, B, C, and D, the evidence from RCTs already provides high certainty and perfectly answers the question, including with regards to applicability, hence looking for NRSI will be unnecessary because the high certainty will not be improved. Other scenarios (e.g., cells E, I, M) are highly unlikely to occur for benefits (although they are plausible for adverse outcomes when large effects in RCTs are imprecise) and looking for NRSI may rarely be informative in these situations or will require individual case assessment of the reasons for lower certainty in RCTs. In other cases (cells F, G, H, J, K, L of Fig. 2) looking for NRSI may be informative, but individual case assessments of reasons for lower certainty in RCTs are required —e.g., indirectness in RCTs can be mitigated by NRSI. When RCTs provide very low certainty in the evidence, looking for NRSI may be useful as in cells M, N, O, P (although situations M and N are less likely to occur, and for P, evidence from NRSI would not increase the certainty).

### 4.2 Future guidance for using randomized and non-randomized studies in systematic reviews and health guidelines

In systematic reviews that include RCTs and NRSI, evidence from both type of studies can be presented either separately as narrative syntheses (with tables summarizing the evidence from RCT and NRSI), as quantitative analyses (in separate meta-analyses or a single pooled estimate), or a combination of both. In a recent survey we asked authors of systematic reviews about their preferences when facing a research question that could be informed by RCTs and NRSI; 17.5% preferred combining RCTs and NRSI into a single pooled estimate (i.e., in meta-analyses), while a majority reported their findings separately for the two types of study designs — either in sub-groups, in separate meta-analyses, or in narrative tables [6]. The issue of integration (using the 2 types of evidence in any of these forms) and the options for portraying both types of study will be discussed in depth in subsequent GRADE guidance.

When crafting health recommendations, guideline development groups or panelists must decide if using the two bodies of evidence would leave them with higher certainty in the evidence than if they would use only 1 of the bodies of evidence, considering each GRADE domain affected and the implications on the final recommendation per outcome.

## 5 The role of ROBINS-I

**Fig. 3:**
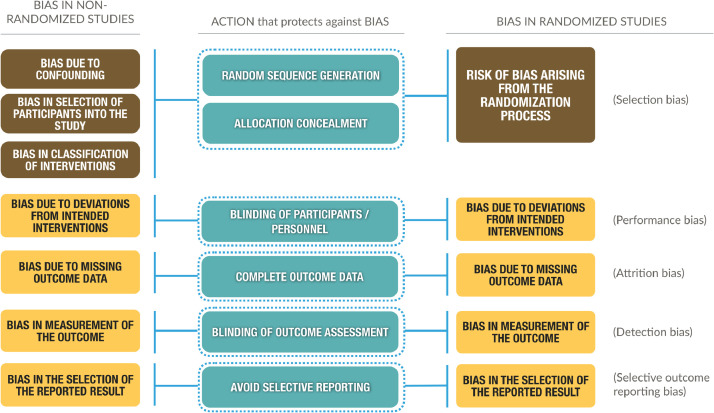
Types of bias met in non-randomized studies (left column) based on the ROBINS-I tool and randomized studies (right column) based on the RoB 2.0 tool, with the situations or actions that, when properly performed (center column), protects against these biases in RCTs and *would* prevent bias in NRSI if we were able to do a random assignment of participants; this is the hallmark feature of the ‘target trial’. To the right, in parentheses, are depicted other nomenclatures for biases, which are based on the classic (previous) risk of bias tool from Cochrane.

Until now, we have assessed the integration of both bodies of evidence in GRADE irrespective of which risk of bias assessment tool had been used. Several tools to assess risk of bias in NRSI exist (e.g., Newcastle-Ottawa, EPIQ, CASP, ROBINS-I) [18], [19], [20] and GRADE does not require the use of a specific instrument as long as the instrument is suitable for the purpose and the assessment of risk of bias transparent. Among these, ROBINS-I [5,6] (Fig. 3) represents a leeway to understand the similarities between RCTs and NRSI more than their differences. We previously described the impact of using ROBINS-I in GRADE in detail [6].

A useful contribution of the ROBINS-I tool, within the framework of developing a systematic review, is the conceptualization and consideration of a “target trial” (i.e., a hypothetical, large, pragmatic RCT that assesses the effect of the same intervention in the same population), which prompts authors assessing a clinical question about an intervention to ask, how a study that answers this question would be conducted by a randomized controlled experiment, regardless of the feasibility to do it. The target trial, in terms of the integration of NRSI with RCTs, facilitates the comparison between RCT and NRSI because they are placed on a common metric allowing the investigator to evaluate bias in the NRSI compared to the target trial.

The main difference between RCTs and NRSI results from randomization that protects essentially against imbalances in prognostic factors [6]. In ROBINS-I, a low risk of bias NRSI would be considered equal to a well conducted RCT. If the assumption that NRSI have none or minimal concerns of bias due to confounding and selection of participants holds —e.g., in an (ideally) well conducted interrupted time series — there should be no major concerns when such NRSI are integrated with RCT, especially if other GRADE domains are similar and effect estimates are coherent. However, we have not yet identified an example in which this is the case. Studies assessed with ROBINS-I may also yield high certainty evidence if other classical upgrading domains apply (e.g., if very large effects are present) [6]. This and other issues are still debated and will be discussed and presented in future GRADE guidance.

## 6 Summary and next steps

Including both RCTs and NRSI in a single systematic review or health guideline has generated controversy and diverse opinions [21]. GRADE can guide authors of evidence syntheses in considering RCTs and NRSI to inform health questions. In some situations, review teams will decide not to search for NRSI to address issues of relative effects, for instance, when they anticipate identifying large well-conducted RCTs evaluating the efficacy of an intervention. Under such circumstances, searching, screening, analyzing, and presenting evidence from NRSI will unnecessarily add substantial work. Yet, on occasions it may be desirable to search other sorts of NRSI that can inform specific issues such as baseline risks for an outcome in people not receiving an intervention.

Practitioners, coverage decision makers, health policymakers, and other stakeholders often face challenging health questions for their decisions and recommendations. These questions require evidence syntheses that strive for the highest certainty of evidence, whether this comes from high certainty RCTs or from NRSI that further complement (e.g. when indirect or imprecise) or replace the body of evidence from RCTs (if the evidence from NRSI is of higher overall certainty than RCTs).

In this article, we provide guidance for authors interested in maximizing the amount of informative evidence in health syntheses from different study designs. In subsequent work, we will address the issue of using both RCTs and NRSI in systematic reviews using GRADE, including the question of whether or not to pool them, and if they can be pooled, what conditions should be fulfilled. Meanwhile, further research is needed to address the distribution of GRADE certainty of evidence levels in systematic reviews that includes RCTs and NRSI, or which GRADE domains prove to have serious limitations when review authors consider both bodies of evidence.

## 7 Summary points

• The GRADE approach supports authors in deciding whether to search for and integrate NRSI with RCT in evidence syntheses about health interventions.
• If authors identify RCTs that prove to have ***high*** certainty evidence for critical and important outcomes, we suggest not screening, nor using NRSI for an evidence synthesis.
• With ***moderate*** certainty of evidence from RCTs, it is unlikely that NRSI will supply higher certainty than moderate. NRSI will be classified as high or moderate only when authors can show reasons for rating up (typically –very– large effects, dose response relationships or opposing plausible residual confounding). However, NRSI may serve as complementary or sequential evidence when the reason to downgrade RCTs to moderate is due to indirectness.
• When authors anticipate or identify ***low*** certainty evidence from RCTs for critical or important outcomes, in particular undesirable health outcomes, searching for relevant NRSI may allow drawing conclusions with more confidence if they have information suggesting well-done NRSI are available (i.e., NRSI that may complement or be used in sequence to RCTs)
• When authors anticipate or identify ***very low*** certainty evidence from RCTs for critical or important outcomes, in particular undesirable health outcomes, they should also search for relevant NRSI (i.e., NRSI may complement, replace, or be used in sequence to RCTs)

## Article history

• Survey Slides and case studies presented at GRADE meetings in Washington, D.C., U.S. (2016), Seoul, South Korea (2016), Rome, Italy (2017), and Hamilton, Canada (2017).
• Article first draft on 31-May-2017.
• Reviewed by Holger Schunemann, Gordon Guyatt, and Jan Brozek on 27-July-2017.
• Presented as part of CC's Ph.D. Thesis on 28-September-2017
• Presented in Bogota, Colombia on April 2018.
• Revised September, October, November, and December 2018
• Revised September 2019
• Revised December 2019 after calls
• Revisions by CC until June 2020
• Revised by HJS June 2020; January 2021
• Approved by GGG, March 2021

## Author's contributions

Carlos Cuello

***Conceptualization and writing of original draft***

Carlos A. Cuello-Garcia; Gordon Guyatt; Holger J. Schünemann

***Writing, review, editing, final approval***

Carlos A. Cuello-Garcia; Nancy Santesso; Rebecca L. Morgan; Jos Verbeek; Kris Thayer; Mohammed T. Ansari; Joerg Meerpohl; Lukas Schwingshackl; Srinivasa Vittal Katikireddi; Jan L. Brozek; Barnaby Reeves; Mohammad H. Murad; Maicon Falavigna; Reem Mustafa; Deborah L. Regidor; Paul Elias Alexander; Paul Garner; Elie A. Akl; Gordon Guyatt; Holger J. Schünemann

## Acknowledgments and Funding

CCG, NS, RLM, JV and HJS have received funding from the Methods Innovation Fund from Cochrane for the development of this guidance.

SVK acknowledges funding from an NRS Senior Clinical Fellowship (SCAF/15/02), the Medical Research Council (MC_UU_00022/2) and the Scottish Government Chief Scientist Office (SPHSU17).

Part of this work has been presented in scientific conferences and at GRADE working group meetings and Cochrane symposia.We are thankful to all Cochrane, G.I.N., and GRADE members for their support and advice throughout this project, as well all McMaster graduate students, faculty, and staff.

The Cochrane Methods Innovation grant, the National Toxicology Program in the U.S., and the McMaster GRADE centre also provided support for this project.

## Footnotes

1 We will use the term “estimates of relative effects”. The reader can assume we are referring to estimates of relative effect of interventions on binary outcomes or absolute effect from studies using continuous variables.
